# Supplementary material for: Molecular Investigation of Recent Canine Parvovirus-2 (CPV-2) in Italy Revealed Distinct Clustering
Source: Viruses. 2022 Apr 28;14(5):917. doi: 10.3390/v14050917 (PMC9143876; doi:10.3390/v14050917)
Supplement: Supplementary file 1 [file viruses-14-00917-s001.zip › viruses-1684140-supplementary.pdf]

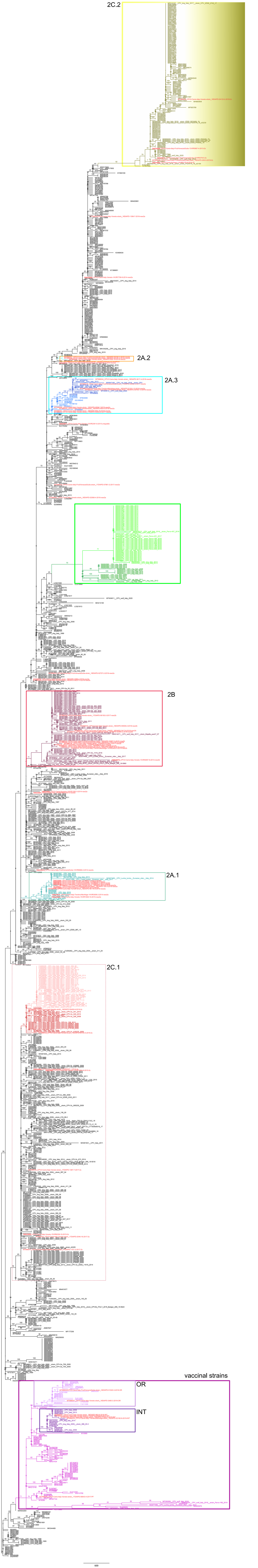
2C.2
2A.2
2A.3
2B
2A.1
2C.1
vaccinal strains
OR
INT

Figure S1. Phylogenetic tree of the VP2 nucleotide region of CPV-2 identified in the present study (highlighted in red) and similar CPV-2 strains available in GenBank. In rectangular boxes are reported eight monophyletic groups containing the majority of the IZSVe CPV-2 strains.
